# Genomic Analysis of *Bacillus megaterium* NCT-2 Reveals Its Genetic Basis for the Bioremediation of Secondary Salinization Soil

**DOI:** 10.1155/2020/4109186

**Published:** 2020-02-28

**Authors:** Bin Wang, Dan Zhang, Shaohua Chu, Yuee Zhi, Xiaorui Liu, Pei Zhou

**Affiliations:** ^1^School of Agriculture and Biology, Shanghai Jiao Tong University, Shanghai 200240, China; ^2^The International Peace Maternity and Child Health Hospital, School of Medicine, Shanghai Jiao Tong University, Shanghai, China

## Abstract

*Bacillus megaterium* NCT-2 is a nitrate-uptake bacterial, which shows high bioremediation capacity in secondary salinization soil, including nitrate-reducing capacity, phosphate solubilization, and salinity adaptation. To gain insights into the bioremediation capacity at the genetic level, the complete genome sequence was obtained by using a multiplatform strategy involving HiSeq and PacBio sequencing. The NCT-2 genome consists of a circular chromosome of 5.19 Mbp and ten indigenous plasmids, totaling 5.88 Mbp with an average GC content of 37.87%. The chromosome encodes 5,606 genes, 142 tRNAs, and 53 rRNAs. Genes involved in the features of the bioremediation in secondary salinization soil and plant growth promotion were identified in the genome, such as nitrogen metabolism, phosphate uptake, the synthesis of organic acids and phosphatase for phosphate-solubilizing ability, and Trp-dependent IAA synthetic system. Furthermore, strain NCT-2 has great ability of adaption to environments due to the genes involved in cation transporters, osmotic stress, and oxidative stress. This study sheds light on understanding the molecular basis of using *B. megaterium* NCT-2 in bioremediation of the secondary salinization soils.

## 1. Introduction

Soil application of organic and inorganic fertilizers for crop and vegetable cultivation is the major source for soil nitrate-nitrogen (nitrate-N), which increases agricultural productivity. However, the vegetable yields do not increase continuously with soil nitrate-N [1]. A large accumulation of nitrate in soil results in soil secondary salinization, having various adverse effects on soil productivity, and nitrate accumulation in vegetables [2]. What is more, the reduction of nitrate to nitrite can cause various human diseases [1]. Soil secondary salinization is a severe problem in intensively managed agricultural ecosystems [3]. It is required to develop a low-cost bioremediation method to remove nitrate from soil.

In our previous study, *Bacillus megaterium* NCT-2 was isolated from the secondary nitrate-salinized soil in a greenhouse, which shows high nitrate-reducing capacity and salinity adaptation in secondary salinization soil [4]. It can remove nitrate at initial nitrate-N concentrations ranging from 100 mg/L to 1,000 mg/L and grow well in inorganic salt medium with 4.0% sodium chloride [4]. In our field trails, the concentrations of NO_3_^−^ in both soil and plant were reduced significantly when we used the NCT-2 strain mixed with straw powder to treat secondary salinization soil (unpublished). Moreover, this strain showed significant phosphate-solubilizing ability of insoluble inorganic phosphates in the culture medium [5]. Strain NCT-2 has the potential to be utilized as a biofertilizer for bioremediation of the secondary nitrate-salinized soil and plant growth promotion [6].

The Gram-positive bacterium *Bacillus megaterium* is found in diverse habitats from soil to sediment, sea, and dried food. It was named after its big size with a volume approximately 100 times than that of *Escherichia coli* [7]. Its big size made it ideal to be used in studies of cell structure, protein localization, sporulation, and membranes [8, 9]. Due to no production of endotoxins associated with the outer membrane and no external alkaline proteases, they are used widely as desirable cloning hosts in food and pharmaceutical production processes for *α*- and *β*-amylases in the baking industry [10, 11], penicillin acylase [12–14], and vitamin B12 [15], such as *Bacillus megaterium* DSM 319, *Bacillus megaterium* QM B1551, and *Bacillus megaterium* WSH 002 [16, 17]. The genomes of them have been sequenced to gain insights into the metabolic versatility that facilitate biotechnological applications, not the bioremediation of secondary salinization soil [18, 19].

Despite the previously published work sequenced the 5.68 Mb draft genome of *B. megaterium* NCT-2 by using the Solexa platform, consisting of the 204 contigs, it focused only on the multiple alignments of nitrate assimilation-related gene sequences [20]. The functional nitrate assimilation-related genes (the nitrate reductase electron transfer subunit, the nitrate reductase catalytic subunit, the nitrite reductase [NAD(P)H] large subunit and small subunit, and the glutamine synthetase) were identified [20]. The genes that could be involved in the full potential of strain NCT-2 in the bioremediation of secondary salinization soil remain unknown. For this, we obtained its complete genome sequence by using a multiplatform strategy involving HiSeq and PacBio sequencing. Furthermore, we performed a comprehensive analysis of nitrogen metabolism and plant growth-promoting features. The comparative analysis might be helpful for use in soil bioremediation.

## 2. Methods

### 2.1. DNA Preparation and Genome Sequencing

*B. megaterium* NCT-2, isolated from the secondary salinized greenhouse soil in China, was cultured in a defined inorganic salt medium as previously described [4]. It was registered in China General Microbiological Culture Collection Center under CGMCC No. 4698. Genomic DNA was isolated using QIAGEN DNeasy Blood & Tissue Kit (Hilden, Germany). The concentration and quality of DNA were determined by a Qubit Fluorometer (Thermo Scientific, USA), NanoDrop Spectrophotometer (Thermo Scientific, USA), and agarose electrophoresis. The whole genome of the *B. megaterium* strain NCT-2 was sequenced by the BGI Tech Solutions Co., Ltd. (Shenzhen, China) by using Illumina Hiseq 4000 short-read sequencing platform (Illumina Inc., San Diego, CA, USA) (insert size, 500 bp; 2 × 125 bp read length) and PacBio RSII long-read sequencing platform (Pacific Biosciences of California, Inc., Menlo Park, CA, USA) ([Supplementary-material supplementary-material-1]).

### 2.2. Genome Assembly and Annotation

After quality control, the *de novo* assembly of the whole NCT-2 genome was performed using the RS_HGAP Assembly3 in the SMRT Analysis pipeline version 2.2.0 [21]. The HiSeq clean reads were preliminarily assembled into contigs and then were used for hybrid error correction of the subreads from PacBio. There were two rounds of error correction. One was analyzed by using SOAPsnp and SOAPIndel [22] and another was by using the Genome Analysis Toolkit (GATK) [23]. Finally, SSPACE-LongRead [24] and Celera assemble [25] were used to generate a high-quality genome. The finished NCT-2 genome was submitted to GenBank, replacing the previous version of the draft genome [20].

The protein-coding genes were predicted by using Glimmer 3.02 [26], and the tandem repeats were detected with Tandem Repeat Finder 4.04 [27]. The gene function annotation was accomplished by blasting the protein sequences against the database of Kyoto Encyclopedia of Genes and Genomes (KEGG) [28]. In addition, the RAST web server (https://rast.nmpdr.org) with the default parameters was used to catalog all the predicted genes into subsystems according to functional categories [29, 30]. CGView was used to produce the maps of the circular genomes with gene feature information [31]. Genome alignments with locally collinear blocks were performed with MAUVE [32].

### 2.3. Phylogenetic Analysis

The whole genome-based phylogenetic analysis was performed by using the CVTree 3.0 online server [33, 34]. Fourteen genome sequences were obtained from GenBank. A phylogenetic tree was constructed by the neighbor-joining method using MEGA analysis [35–37]. In addition, FusionDB was used to analyze the functional repertories of *B. megaterium* NCT-2 and identify the nearest “neighbors” based on the functional similarities [38, 39].

## 3. Results and Discussion

### 3.1. General Genomic Characteristics

**Figure 1:**
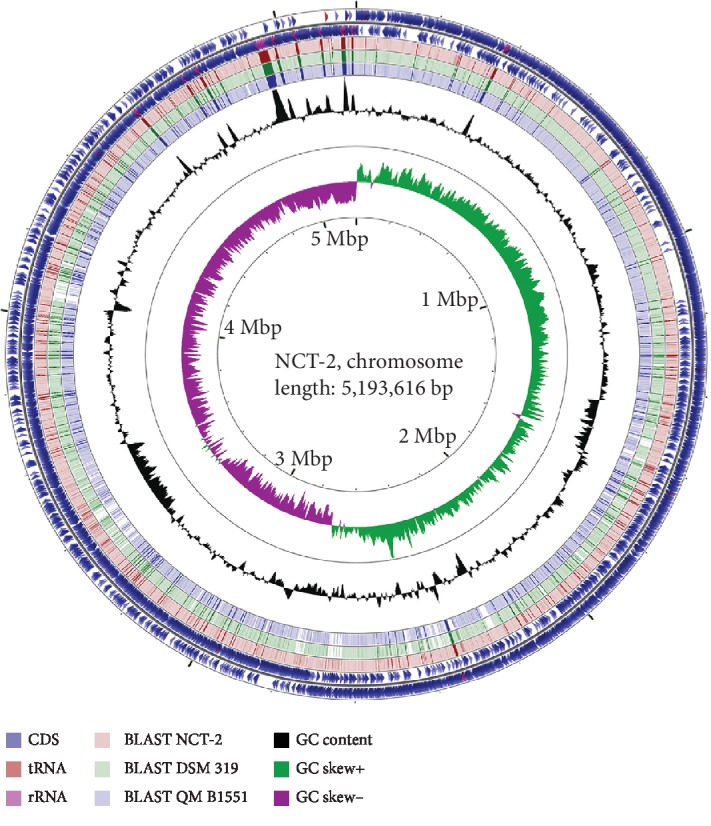
Genetic and physical map of the genome of *B. megaterium* NCT-2 prepared using CGView. Circles from the outside to the inside show the position of protein-coding sequences (blue), tRNA gene (red), and rRNA genes (pink) on the positive (circle 1) and negative (circle 2) strands. Circles 3-5 show the positions of BLAST hits detected through BLASTx comparisons of *B. megaterium* NCT-2 against itself (circle 3), *B. megaterium* DSM 319 (circle 4), and *B. megaterium* QM B1551 (circle 5). Circles 6 and 7 show plots of GC content and GC skew plotted as the deviation from the average for the entire sequence.

A total of ~1,189 Mb raw data and ~1,147 Mb clean data were obtained after filtering the low-quality reads generated by the HiSeq platform. The PacBio platform yielded 48,392 polymerase reads (with the average size of 12.9 kb) and 622 Mb subreads after quality control. The complete genome was assembled by taking advantage of the higher accuracy short reads from the HiSeq platform and the long subreads from the PacBio platform. The genome consists of a circular chromosome of 5.19 Mb with an average GC content of 38.2% (accession number: CP032527.2) and ten circular plasmids designated as the plasmid pNCT2-1 to pNCT2-10 (accession numbers: CP032528.1-CP032537.1). Sequence information was visualized in CG view Server (Figure 1 and [Supplementary-material supplementary-material-1]). The total genome size is 5.88 Mb with an average GC content of 37.87%. The whole genome contains 6,039 genes, including 5,606 coding sequences, 203 RNA genes, and 230 pseudo genes. There are 127 identified tandem repeat sequences (TRF), 83 minisatellite DNA, and 7 microsatellite DNA.

The general features of *B. megaterium* NCT-2 were compared with five genomes of *Bacillus* strains (*Bacillus megaterium* DSM 319, *Bacillus megaterium* QM B1551, *Bacillus subtilis* subsp. subtilis str. 168, *Bacillus cereus* Q1, and *Bacillus licheniformis* DSM 13) (Table 1). The genome GC contents for three *B. megaterium* strains are around 38%. Strain NCT-2 has the largest genome size and most coding sequences and RNA genes, such as 53 rRNAs and 142 tRNAs. There were 14 rRNA operons on the negative chain and one rRNA operon on the positive strand with a 16S-23S-5S organization. In addition, the positive chain had one unusual rRNA operon with a 16S-23S-5S-5S organization and a single 5S rRNA. The microbial genome size is positively correlated with their environment adaptability [40]. One typical characteristic of soil microorganisms is the high number of rRNAs, which is helpful for fast growth, successful sporulation, germination, and rapid response to changing the availability of nutrients [41–44]. These features indicate that strain NCT-2 has great ability of adaptation to various environments.

Most strains of *Bacillus megaterium* carry multiple plasmids, such as strain QM B1551 has seven resident plasmids [18], *Bacillus megaterium* strain 216 has ten plasmids [45], and *Bacillus megaterium* NBRC 15308 has six plasmids. As for the ten plasmids in strain NCT-2, the sizes range from 9,625 bp to over 132 kb making up 11.7% of the whole genome ([Supplementary-material supplementary-material-1]). The plasmids have significantly lower GC contents than the chromosome (33.7-37.0% versus 38.2%). There are 761 coding sequences and 23 RNA genes. Both plasmids pNCT2-2 and pNCT2-6 had one tRNA. In addition, pNCT2-7 had 18 tRNAs, one 5S RNA, one large subunit ribosomal RNA (LSU rRNA), and one small subunit ribosomal RNA (SSU rRNA). Additional rRNA operons carried on plasmids slowed the growth rates of *E. coli* on poor carbon sources [46]. Further investigations are needed to clarify the role of plasmids in bacterial growth and adaptations to high-nitrate environments in bioremediation of the secondary salinization soils.

### 3.2. Phylogenetic Lineage Analysis

**Figure 2:**
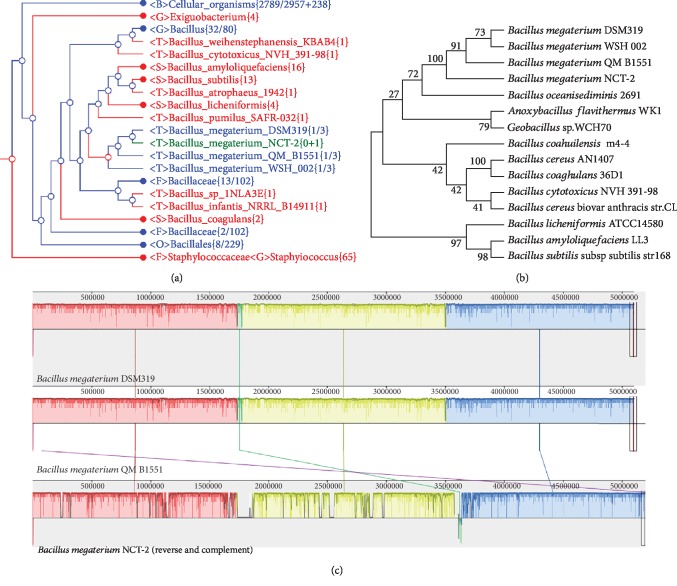
Phylogenetic tree showing the position of *Bacillus megaterium* NCT-2. (a) The tree was constructed based on the frequency of >6-string predicted peptides from the whole genome sequences by using CVTree 3.0. (b) The tree is based on 16S rDNA phylogenetic analysis by using MEGA7 with the neighbor-joining method. The bootstrap consensus tree inferred from 1,000 replicates is taken to represent the evolutionary history of the taxa analyzed. (c) Chromosomal similarity among strain NCT-2, DSM 319, and QM B1551 by using Mauve alignments. Three local collinear blocks (LCBs) on the chromosomes were identified and joined by connecting lines in the three genomes.

We used CVTree 3.0 to construct a phylogenetic tree based on the complete proteomes with *Macrococcus caseolyticus* JCSC5402 as an outgroup. The obtained tree (Figure 2(a)) indicated that *B. megaterium* NCT-2 was most homologous to *B. megaterium* DSM 319 and then *B. megaterium* QM B1551. Similarly, genome comparison using the RAST Prokaryotic Genome Annotation Server also showed that the genomic sequence of NCT-2 had a higher comparison score with *B. megaterium* QM B1551 and *B. megaterium* DSM 319 ([Supplementary-material supplementary-material-1]). Furthermore, 16S rDNA sequences from 15 *Bacillus* strains were used to construct a phylogenetic tree by MEGA7 with the neighbor-joining method. The neighbor-joining phylogenetic tree shows that strain NCT-2 is closest to *B. megaterium* QM B1551, *B. megaterium* DSM 319, and *B. megaterium* WSH 002 (Figure 2(b)). Whole-genome alignment of *B. megaterium* NCT-2 to closely related QM B1551 and DSM 319 by using MAUVE revealed that the chromosomes of the three strains showed overall collinearity (Figure 2(c)).

### 3.3. Functional Annotations of *B. megaterium* NCT-2

To investigate the function of the 5,606 coding sequences, the GO database, the KEGG database, the COG database, and RAST web server were used. The 3,159 genes annotated by GO were classified into biological processes, cellular components, and molecular functions ([Supplementary-material supplementary-material-1]). The top five categories were catalytic activity (1,822), metabolic process (1,786), cellular process (1,567), single-organism process (1,400), and binding (1,214).

**Figure 3:**
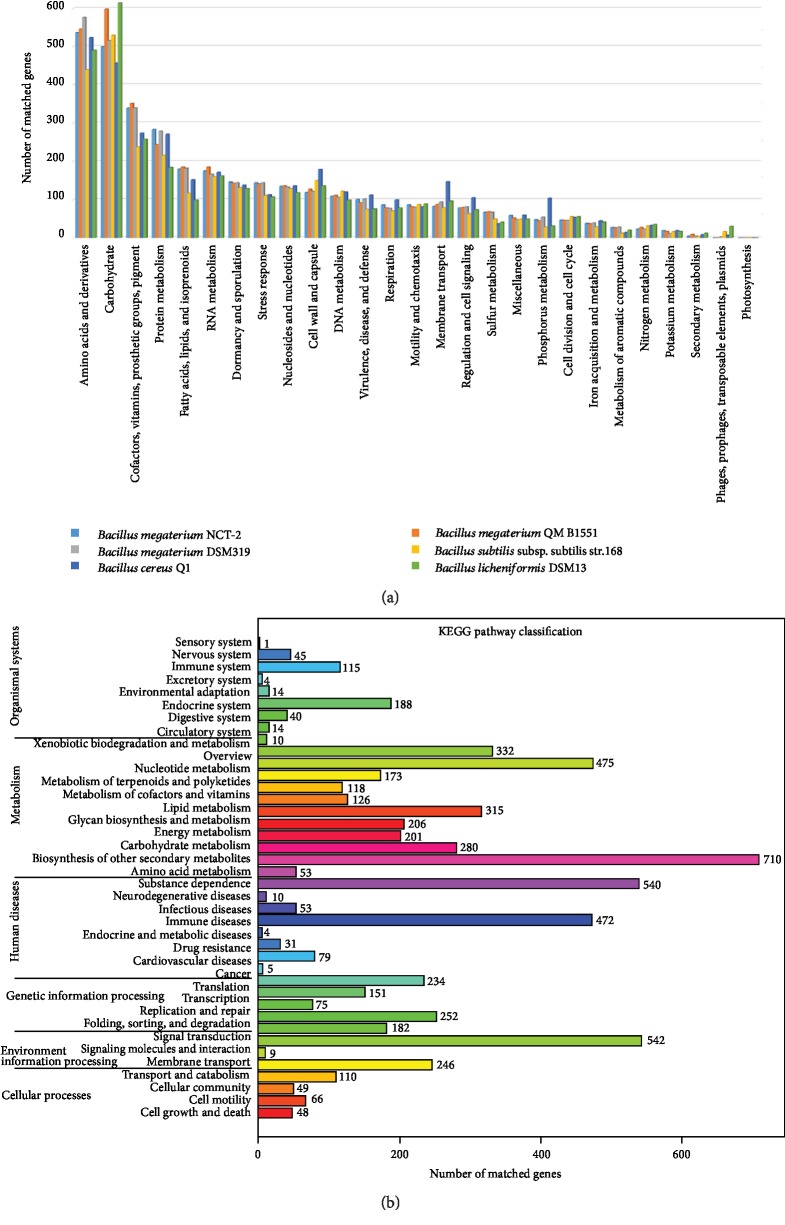
Distribution of genes based on Cluster of Orthologous Groups (COG) classification and KEGG pathways in *B. megaterium* NCT-2. Only genes assigned to the COG and KEGG categories were used for analysis. (a) Functional categorization of *B. megaterium* NCT-2 compared with 5 related *Bacillus* strains based on the COG database. (b) KEGG classification of *B. megaterium* NCT-2.

2,338 chromosomal genes (44%) were assigned into 477 subsystems by RAST ([Supplementary-material supplementary-material-1]). Subsystem category comparisons among six related *Bacillus* strains showed that the number of genes involved in “Amino Acids and Derivatives” and “Carbohydrates” was highest in the genome of the six strains (Figure 3(a)). In addition, *Bacillus megaterium* has more genes involved in “Cofactors, Vitamins, Prosthetic Groups, Pigments.” The top five categories in strain NCT-2 were the “Amino Acids and Derivatives” (538), “Carbohydrates” (500), “Cofactors, Vitamins, Prosthetic Groups, Pigments” (340), “Protein Metabolism” (283), and “Fatty Acids, Lipids, and Isoprenoids” (180).

Likewise, 2,962 genes annotated by the KEGG database were assigned to 38 pathways (Figure 3(b)). The top five enriched pathways were “Biosynthesis of other secondary metabolism” (710), “Signaling molecules and interaction in Environmental information processing” (542), “Substance dependence” (540), “Nucleotide metabolism” (475), and “Immune disease” (472).

Like most strains of *B. megaterium*, which carry more than four plasmids, strain NCT-2 harbors ten indigenous plasmids. Only 75 genes (10%) were assigned into 37 subsystems by RAST ([Supplementary-material supplementary-material-1]), including genes for riboflavin metabolism, butanol biosynthesis, and xylose utilization, and parts of genes in benzoate degradation and metabolism of central aromatic intermediates. There are also genes for cobalt-zinc-cadmium resistance, oxidative stress, and nitrosative stress.

### 3.4. Microbial Functional Similarities

**Figure 4:**
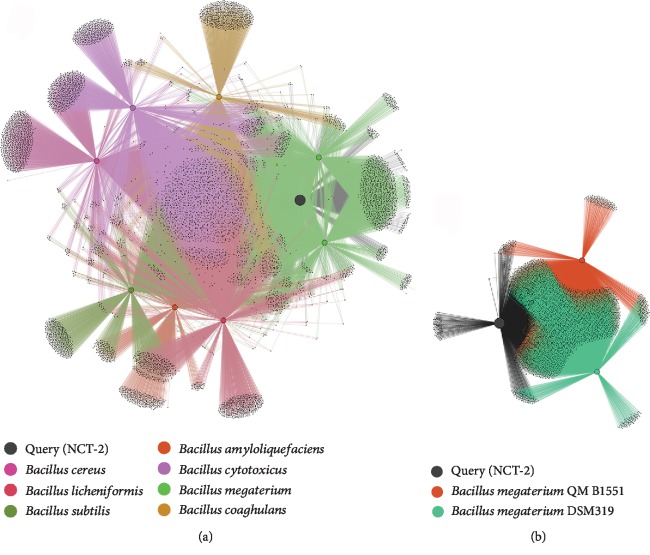
Functional similarity networks analysis by fusion+. (a) Functional similarity network among 9 strains of *Bacillus*. (b) Functional similarity network among 3 strains of *Bacillus megaterium*. The networks contain nodes of organisms and functions. The colored organism nodes are connected to black function nodes.

The translated protein sequence of *B. megaterium* NCT-2 was downloaded from RAST and submitted to the FusionDB web server (https://services.bromberglab.org/fusiondb/mapping) [38]. The submitted proteome (containing 5,364 proteins) matched to 3,662 FusionDB functions, while 228 proteins could not be mapped to any function in their database. The functional similarities of *B. megaterium* NCT-2 with 1,374 taxonomically distinct bacteria (with similarity > 40%) were shown in [Supplementary-material supplementary-material-1], most of them were soil bacterium. Strain NCT-2 is most functionally similar to *B. megaterium* DSM 319 (90%) and *B. megaterium* QM B1551 (89%). The functional relationships among nine *Bacillus* strains were demonstrated by the fusion+ networks (Figure 4(a)). There were 1,290 functions shared by all of them. The common functional annotations related to nitrogen metabolism were nitrite transporter NirC, nitrogen-fixing NifU domain protein, nitroreductase, nitrate transport protein, and 2-nitropropane dioxygenase. Notably, there are 3,047 functions shared among three strains of *B. megaterium* (strain NCT-2, strain QM B1551, and strain DSM 319) (Figure 4(b)). Strain NCT-2 has most of the core genes and pathways, including vitamin biosynthesis and nitrogen metabolism. The nitrogen metabolism-related genes, such as those encoding nitrate transport protein, nitrate/nitrite sensor protein, nitric oxide reductase activation protein, nitrite reductase [NAD(P)H] large subunit, nitrite reductase [NAD(P)H] small subunit, nitrite transporter, nitrite-sensitive transcriptional repressor, nitrogen regulatory protein P-II, nitrogen-fixing NifU domain protein, nitroreductase, and nitroreductase family protein, were located on the chromosome of the three strains. Furthermore, only strain NCT-2 carries the gene encoding for periplasmic nitrate reductase.

### 3.5. Genome Inventory for Nitrogen Metabolism

**Figure 5:**
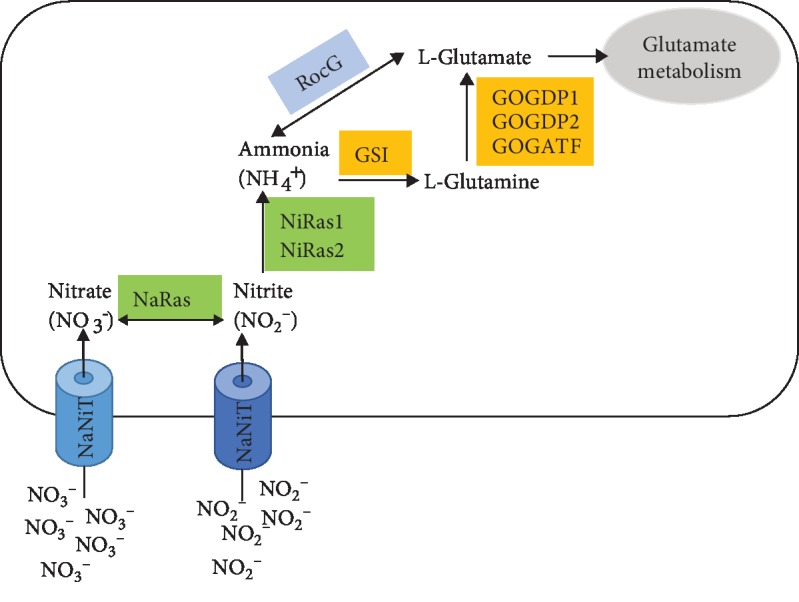
The proposed nitrogen metabolism in *B. megaterium* NCT-2. NaNiT: nitrate/nitrite transporter; NaRas: assimilatory nitrate reductase large subunit (EC 1.7.99.4); NiRas1: nitrite reductase [NAD(P)H] large subunit (EC 1.7.1.4); NiRas2: nitrite reductase [NAD(P)H] small subunit (EC 1.7.1.4); GSI: glutamine synthetase type I (EC 6.3.1.2); GOGDP1: glutamate synthase [NADPH] large chain (EC 1.4.1.13); GOGDP2: glutamate synthase [NADPH] small chain (EC 1.4.1.13); GOGATF: Ferredoxin-dependent glutamate synthase (EC 1.4.7.1); RocG: NAD-specific glutamate dehydrogenase (EC 1.4.1.2).

In our field experiment, strain NCT-2 shows high nitrate-reducing capacity in secondary salinization soil (unpublished). The functional nitrate assimilation-related genes that are involved in the process of converting nitrate to glutamine have been identified [20]. The genes encoding nitrate and nitrite reductase were cloned and overexpressed in *Escherichia coli* [47]. Here, the whole genomic analysis also revealed the genes encoding sensor, transporter, and enzymes are involved in nitrogen metabolism. The genes were scattered in the chromosome. Genes encoding nitrite-sensitive transcriptional repressor (NsrR), which is directly sensitive to nitrosative stress, were found in both the chromosome and the plasmid ([Supplementary-material supplementary-material-1] and [Supplementary-material supplementary-material-1]). *B. megaterium* NCT-2 possessed nitrate/nitrite sensor protein (NaNiS) and nitrate/nitrite transporter (NaNiT) for sensing and transporting the NO_3_^−^ and NO_2_^−^. In the process of nitrate and nitrite ammonification, assimilatory nitrate reductase (NaRas) and nitrite reductase (NiRas) catalyzed the reduction of nitrate to ammonia through nitrite [48]. Then, ammonia was assimilated into amino acids through L-Glutamine and L-Glutamate by glutamine synthetase type I (GSI), Ferredoxin-dependent glutamate synthase (GOGATF), glutamate synthase [NADPH] large chain (GOGDP1), and glutamate synthase [NADPH] small chain (GOGDP2). Ammonium transporter (Amt) was also encoded in the genome. Ammonium is an important nitrogen source for plant growth. Environmental NH_4_^+^/NH_3_ was imported across membranes by Amt for cell growth in prokaryotes and plants [49]. Bacterial Amt proteins act as passive channels for the uncharged gas ammonia (NH_3_) [50]. It means that *B. megaterium* NCT-2 might scavenge NH_4_^+^/NH_3_ in soil instead of providing. In the face of nitrosative stress, genes encoding nitrite-sensitive transcriptional repressor (NsrR) were found in both the chromosome and the plasmid. NsrR played a pivotal role in the regulation of NirK (nitrite reductase), which was expressed aerobically in response to the increasing concentration of NO_2_^−^ and decreasing pH [51]. However, no functional NirK could be found. Instead, two nitric oxide reductase activation proteins (NorD and NorQ) for denitrifying reductase gene clusters were found but without nitric oxide reductase, making the function of denitrification highly unlikely. Thus, the genome analysis proposed that *B. megaterium* NCT-2 could convert nitrate from secondary salinization soil into biomass through glutamate rather than reduce nitrate to nitrous oxide or dinitrogen, which are lost from the soil (Figure 5). It is an effective bioremediation approach to remove nitrate from soils.

### 3.6. Genes Associated with Plant Growth-Promoting Features

Our previous studies on the plant growth promotion of *B. megaterium* NCT-2 revealed that it could produce organic acids (lactic acid, acetic acid, propionic acid, and gluconic acid) and phosphatase in culture medium, showing significant phosphate-solubilizing ability [5]. Inoculation with *B. megaterium* NCT-2 significantly increased the root fresh weight of maize [6]. The genome of NCT-2 contains genes encoding for glucose 1-dehydrogenase (EC 1.1.1.47) and alkaline phosphatase (EC 3.1.3.1). Glucose dehydrogenase can oxidize glucose to gluconic acid, which is the most frequent organic acid produced by phosphate-solubilizing bacteria [52]. Additionally, the phosphate starvation system for phosphate uptake encoded by *pst*S, *pst*C, *pst*A, and *pst*B was also found in the genome. The phosphate solubilization capacity of strain NCT-2 plays a positive role in promoting plant growth by dissolving unavailable P (PO_4_^3-^) in soil to plant available forms.

Many plant growth-promoting bacteria have the ability to synthesize plant auxins (indole-3-acetic acid, IAA) [53, 54], which is a key regulator for plant growth and development, such as cell division and elongation, lateral root production, and flowering [55]. Large-scale genomic analysis of IAA synthesis pathways suggested that plenty of bacteria could synthesize IAA via multiple incomplete pathways, and Firmicutes genomes had the simplest Trp-dependent IAA synthetic system [56]. According to the KEGG analysis, strain NCT-2 could assimilate tryptophan (Trp) ([Supplementary-material supplementary-material-1]) but had incomplete Trp-dependent IAA synthesis pathways, such as the indole-3-acetamide (IAM) pathway and indole-3-pyruvate (IPA) pathway ([Supplementary-material supplementary-material-1]). It had aldehyde dehydrogenase (NAD^+^) (EC 1.2.1.3) and amidase (EC 3.5.1.4) catalyzing the final step of IAA synthesis. However, we could not find the enzymes which convert Trp into IAM and IPA. These results suggested that strain NCT-2 might synthesize IAA from intermediates.

Both the phosphate solubilization and IAA synthesis play important roles in plant growth promotion of strain NCT-2 during biocontrol and bioremediation of the secondary salinization soils.

### 3.7. Genes Involved in Stress Response

*B. megaterium* NCT-2 showed high salinity adaptation in secondary salinization soil in our previous study [4]. From the genome perspective, we can see genes involved in cation transporters (magnesium transport and copper transport system) and stress response, such as osmotic stress, oxidative stress, and detoxification. Glycine betaine, a very efficient osmoprotectant, can be synthesized or acquired from exogenous sources [57]. There are glycine betaine ABC transport systems (*opu*A, *opu*C, and *opu*D) for choline uptake and genes for the glycine betaine biosynthetic enzymes (choline dehydrogenase, *gbs*B, and betaine-aldehyde dehydrogenase, *gbs*A) in strain NCT-2 genome. Moreover, the genome contains genes encoding for superoxide dismutase (EC 1.15.1.1), catalase (EC 1.11.1.6), and ferroxidase (EC 1.16.3.1), protecting bacteria from oxidative stress. It implied that NCT-2 has great ability of adaption to environments.

## 4. Conclusion

A hybrid approach with multiple assembler was used to assemble the complete genome of *B. megaterium* NCT-2. The deeper investigation identified clues associated with the features of the bioremediation of secondary salinization soil and plant growth promotion at the gene level, such as nitrogen metabolism, phosphate uptake, synthesis of organic acids and phosphatase for phosphate-solubilizing ability, and Trp-dependent IAA synthetic system. Furthermore, the genes involved in cation transporters, osmotic stress, and oxidative stress implied that NCT-2 has great ability of adaption to environments. In summary, these results provide valuable genomic resources for further studies and applications of using *B. megaterium* NCT-2 in bioremediation processes of secondary salinization soil.

## Figures and Tables

**Table 1:** General genome features of *B. megaterium* NCT-2 compared with other five *Bacillus* strains.

Strain	*B. megaterium* NCT-2	*B. megaterium* QM B1551	*B. megaterium* DSM 319	*B. subtilis* 168	*B. cereus* Q1	*B. licheniformis* DSM 13
Genome size (Mb)	5.88	5.52	5.10	4.22	5.51	4.22
Chromosome size (Mb)	5.19	5.10	5.10	4.22	5.21	4.22
G+C content (%)	37.8	37.97	38.1	43.5	35.5	46.2
Chromosomal G+C content (%)	38.2	38.3	38.1	43.5	35.6	46.2
Gene number	6039	5674	5245	4536	5856	4382
Coding sequence number	5606	5379	4941	4237	5513	4219
RNA gene number	203	182	153	116	137	98
rRNA genes (5S, 16S, and 23S)	53 (19, 17, 17)	37 (13, 12, 12)	33 (11, 11, 11)	30 (10, 10, 10)	39 (13, 13, 13)	21 (7, 7, 7)
tRNA gene number	142	137	114	86	93	72
Plasmid number	10	7	0	0	2	0

## Data Availability

All data generated or analyzed during this study are included in this published article and its supplementary information files. The genome sequence of *B. megaterium* NCT-2 has been deposited in GenBank. The accession number for the *B. megaterium* NCT-2 chromosome is CP032527.2, and those for ten plasmids are CP032528.1, CP032529.1, CP032530.1, CP032531.1, CP032532.1, CP032533.1, CP032534.1, CP032535.1, CP032536.1, CP032537.1.

## Supplementary Materials

Supplementary Materials Supplementary 1. Figure S1: whole genome sequencing and assembly workflow. Supplementary 2. Figure S2: circular representation of the ten plasmids of *B. megaterium* NCT-2. Supplementary 3. Figure S3: genome similarity of strain NCT-2. The genome of strain NCT-2 was submitted to the web service RAST and was compared with genomes of other strains. A higher comparison score means higher similarity. Supplementary 4. Figure S4: histogram of GO classifications. The results are summarized in three categories: biological process (blue), cellular component (brown), and molecular function (orange). Supplementary 5. Figure S5: genes connected to subsystems according to functional categories. (a) The subsystems of genes from chromosome. (b) The subsystems of genes from plasmids. Supplementary 6. Figure S6: enzymes involved in the nitrogen metabolism of *B. megaterium* NCT-2 from KEGG. Genes of *B. megaterium* NCT-2 were shown in green boxes. Supplementary 7. Figure S7: enzymes involved in phenylalanine, tyrosine, and tryptophan biosynthesis of *B. megaterium* NCT-2 from KEGG. Genes of *B. megaterium* NCT-2 were shown in green boxes. Supplementary 8. Figure S8: enzymes involved in the tryptophan metabolism of *B. megaterium* NCT-2 from KEGG. Genes of *B. megaterium* NCT-2 were shown in green boxes. Supplementary 9. Table S1. plasmids features of *B. megaterium* NCT-2. Supplementary 10. Table S2. the functional similarities of *B. megaterium* NCT-2 with 1,374 bacterial genomes. Supplementary 11. Table S3. gene cluster involved in nitrogen metabolism of *B. megaterium* NCT-2.
