# Supplementary material for: Genomic Analysis of Bacillus megaterium NCT-2 Reveals Its Genetic Basis for the Bioremediation of Secondary Salinization Soil
Source: Int J Genomics. 2020 Feb 28;2020:4109186. doi: 10.1155/2020/4109186 (PMC7066406; doi:10.1155/2020/4109186)
Supplement: Supplementary Materials — Supplementary 1. Figure S1: whole genome sequencing and assembly workflow. Supplementary 2. Figure S2: circular representation of the ten plasmids of B. megaterium NCT-2. Supplementary 3. Figure S3: genome similarity of strain NCT-2. The genome of strain NCT-2 was submitted to the web service RAST and was compared with genomes of other strains. A higher comparison score means higher similarity. Supplementary 4. Figure S4: histogram of GO classifications. The results are summarized in three categories: biological process (blue), cellular component (brown), and molecular function (orange). Supplementary 5. Figure S5: genes connected to subsystems according to functional categories. (a) The subsystems of genes from chromosome. (b) The subsystems of genes from plasmids. Supplementary 6. Figure S6: enzymes involved in the nitrogen metabolism of B. megaterium NCT-2 from KEGG. Genes of B. megaterium NCT-2 were shown in green boxes. Supplementary 7. Figure S7: enzymes involved in phenylalanine, tyrosine, and tryptophan biosynthesis of B. megaterium NCT-2 from KEGG. Genes of B. megaterium NCT-2 were shown in green boxes. Supplementary 8. Figure S8: enzymes involved in the tryptophan metabolism of B. megaterium NCT-2 from KEGG. Genes of B. megaterium NCT-2 were shown in green boxes. Supplementary 9. Table S1. plasmids features of B. megaterium NCT-2. Supplementary 10. Table S2. the functional similarities of B. megaterium NCT-2 with 1,374 bacterial genomes. Supplementary 11. Table S3. gene cluster involved in nitrogen metabolism of B. megaterium NCT-2. [file 4109186.f1.docx]

**Support Information**

**Genomic analysis of *Bacillus megaterium* NCT-2 reveals its genetic basis for the bioremediation of secondary salinization soil**

**Bin Wang ^1^, Dan Zhang ^1^, Shaohua Chu ^1^, Yuee Zhi ^1^, Xiaorui Liu^2*^, Pei Zhou ^1*^**

^1^ School of Agriculture and Biology, Shanghai Jiao Tong University, Shanghai, 200240, China

^2^ The International Peace Maternity and Child Health Hospital, School of Medicine, Shanghai Jiao Tong University, Shanghai, China

^*^Correspondence should be addressed to Pei Zhou and Xiaorui Liu; [peizhousjtu@163.com](mailto:peizhousjtu@163.com) (Z.P.) and [xiaorui1211@126.com](mailto:xiaorui1211@126.com) (X.L.)


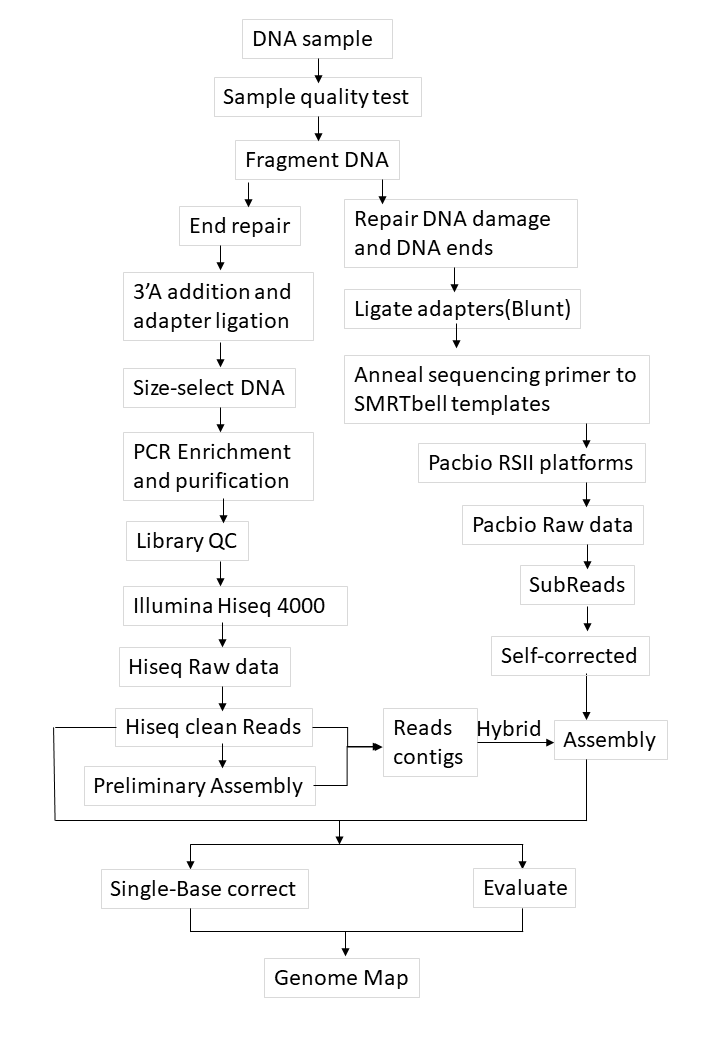


**Figure S1. Whole genome sequencing and assembly workflow.**

**
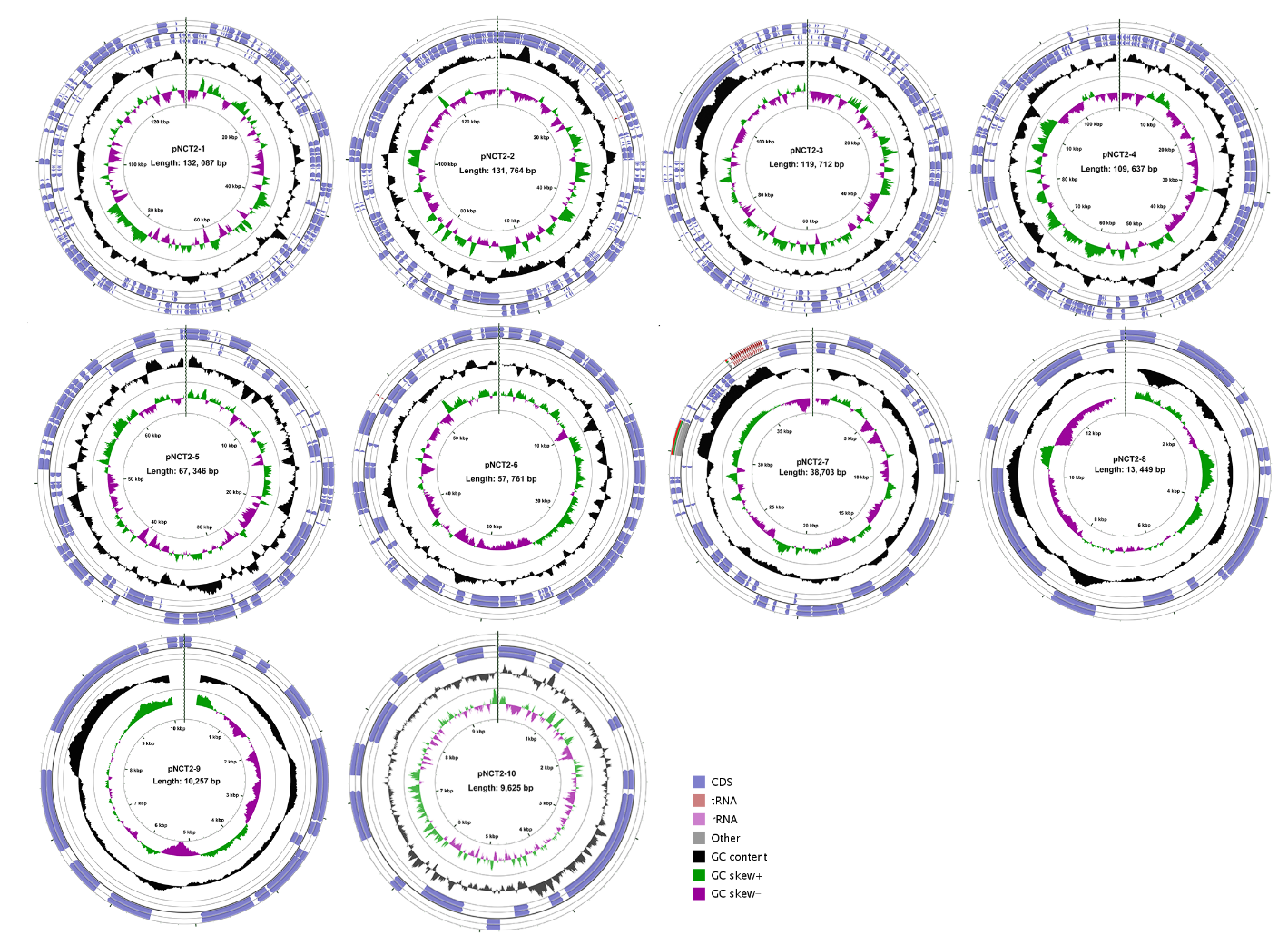
**

**Figure S2. Circular representation of the ten plasmids of *B. megaterium* NCT-2.**

**
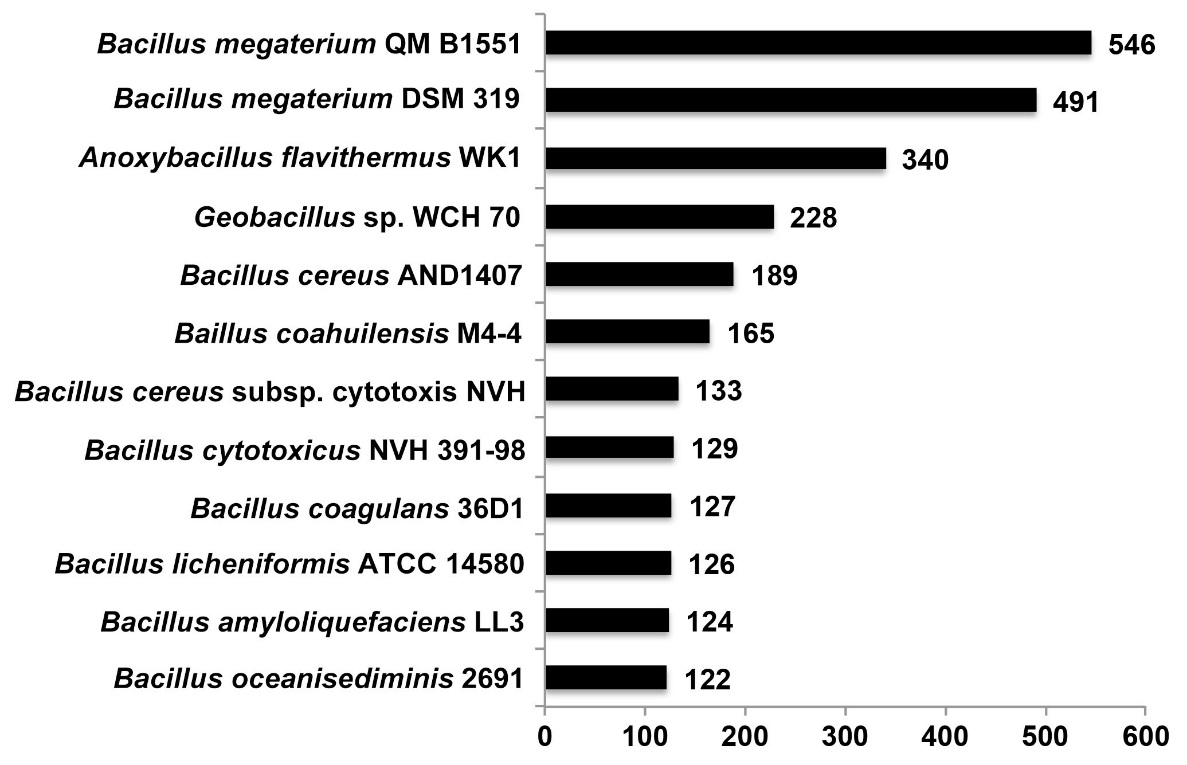
**

**Figure S3. Genome similarity of strain NCT-2.** The genome of strain NCT-2 was submitted to the web service RAST and was compared with genomes of other strains. Higher comparison score means higher similarity.

**
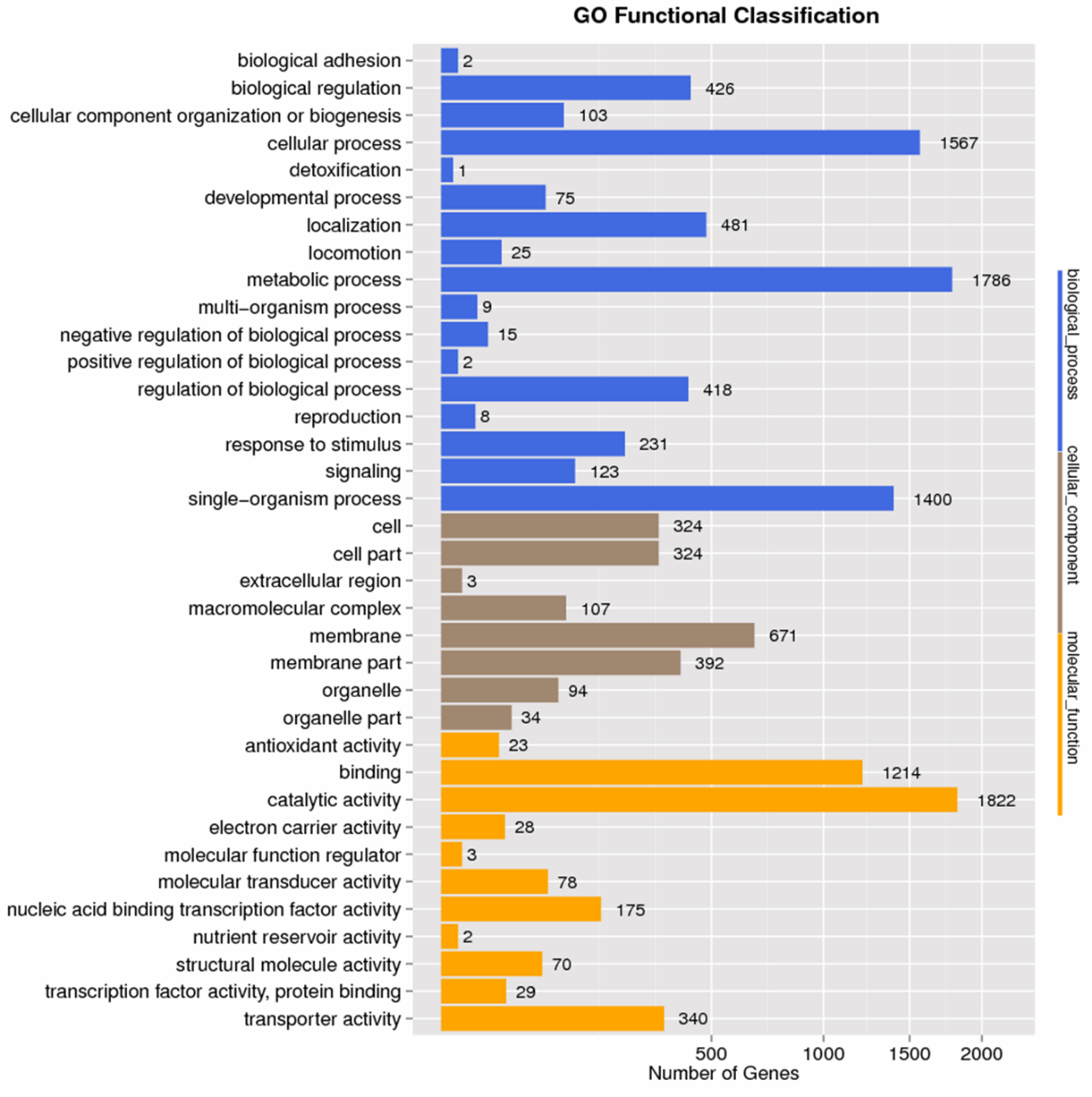
**

**Figure S4. Histogram of GO classifications.** The results are summarized in three categories: biological process (Blue), cellular component (Brown) and molecular function (Orange).

**
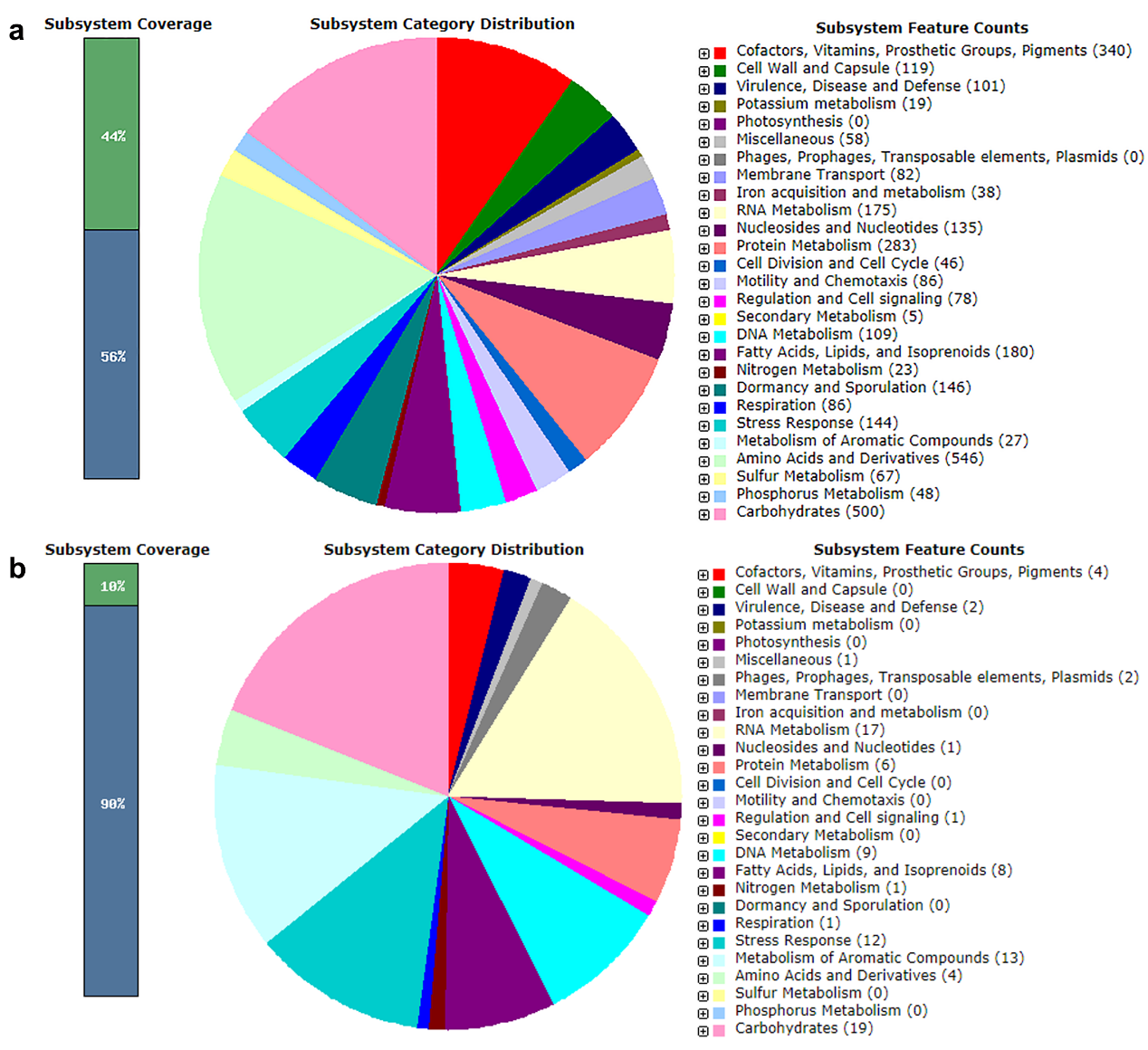
**

**Figure S5. Genes connected to subsystems according to functional categories.** a. The subsystems of genes from chromosome; b. The subsystems of genes from plasmids.**
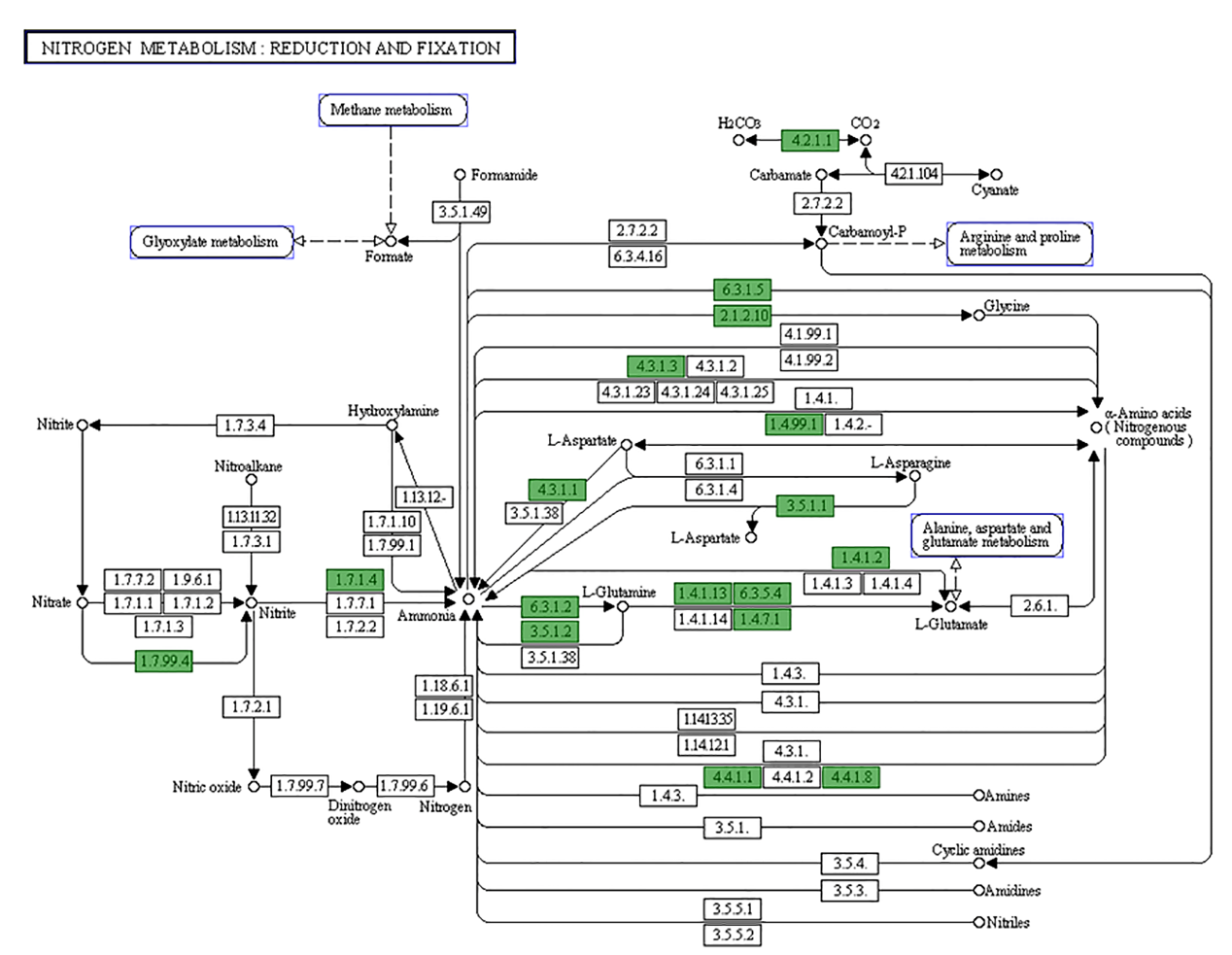
**

**Figure S6. Enzymes involved in the nitrogen metabolism of *B. megaterium* NCT-2 from KEGG.** Genes of *B. megaterium* NCT-2 were shown in green boxes.


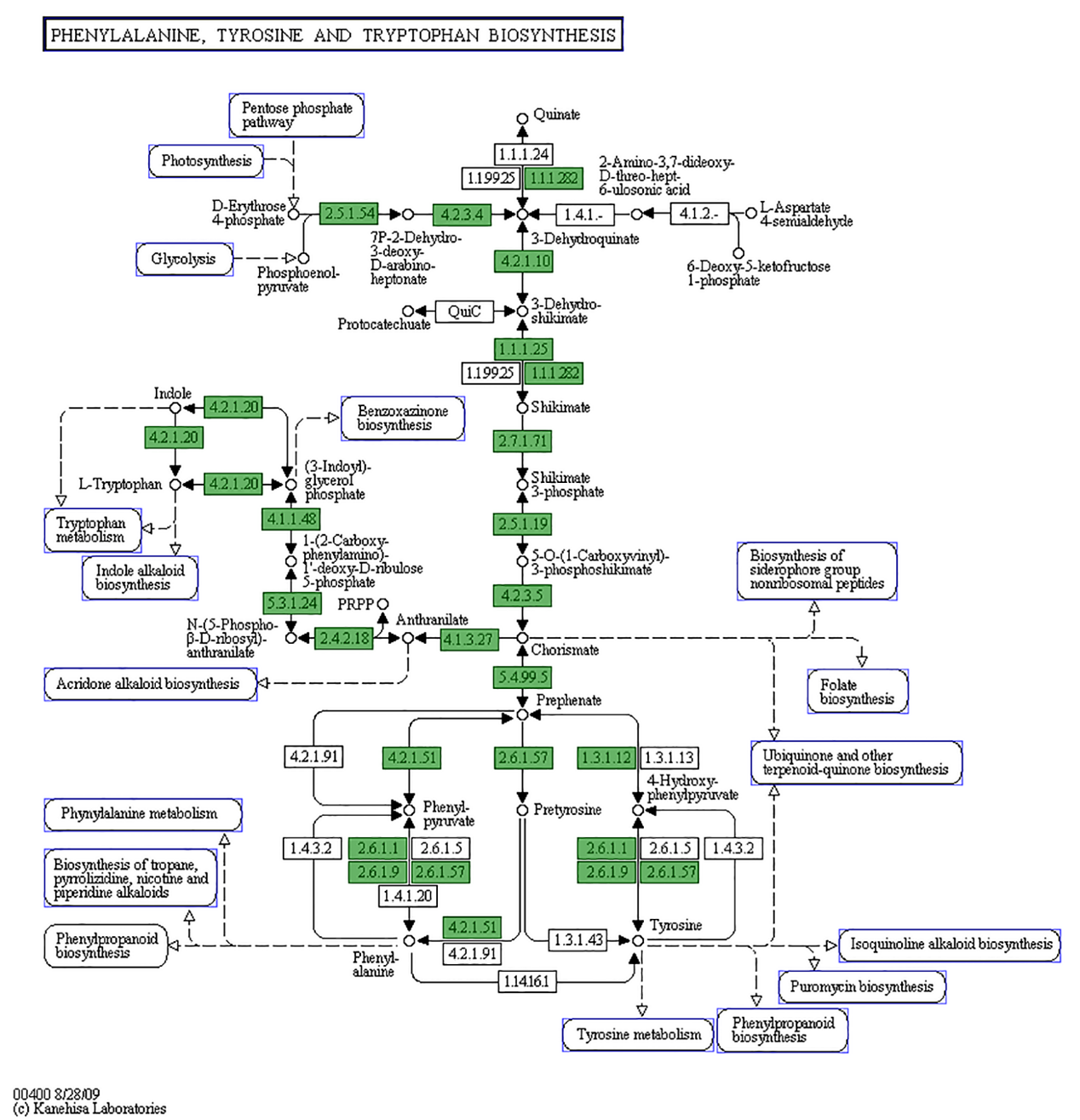


**Figure S7. Enzymes involved in phenylalanine, tyrosine and tryptophan biosynthesis of *B. megaterium* NCT-2 from KEGG.** Genes of *B. megaterium* NCT-2 were shown in green boxes.

**
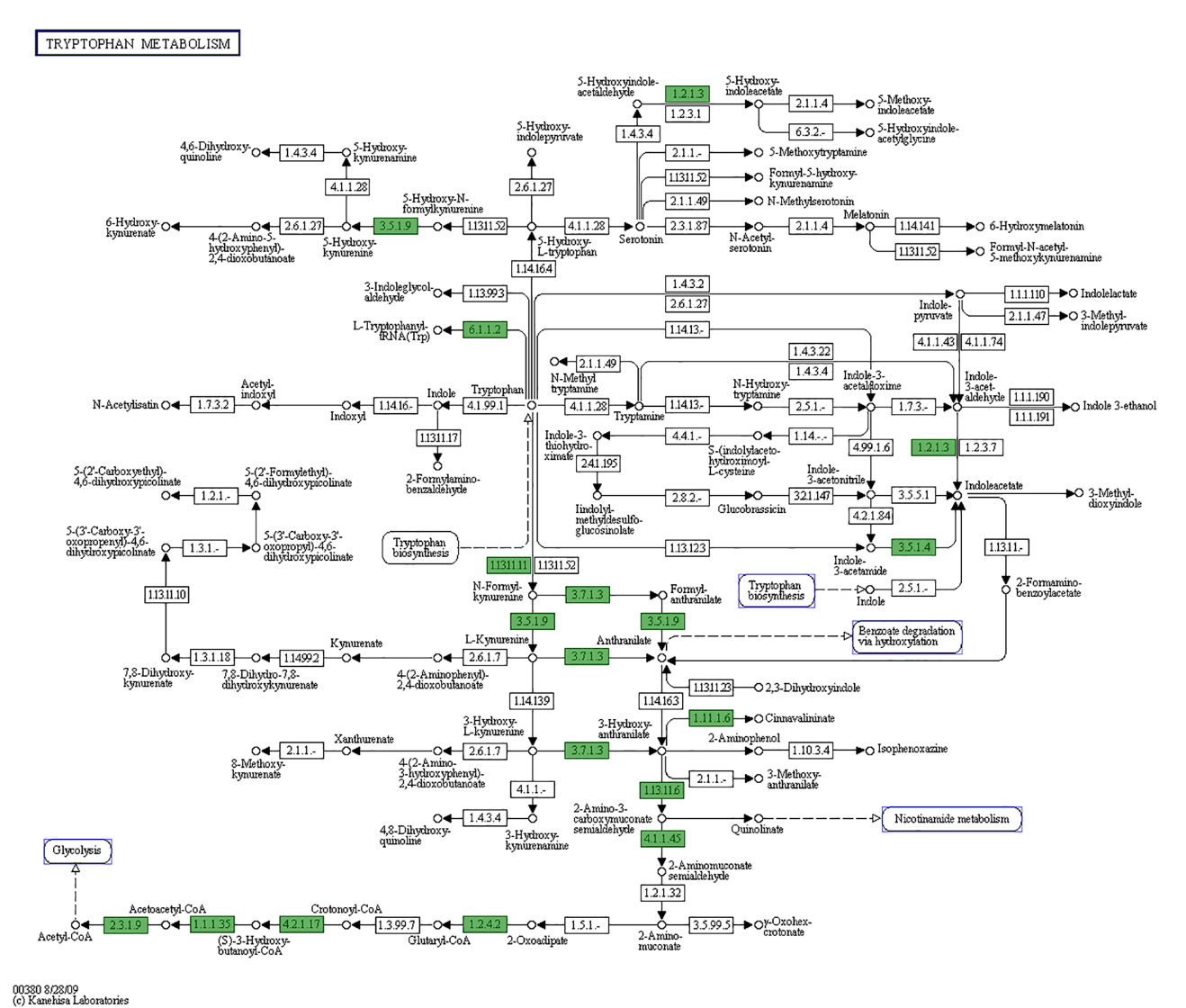
**

**Figure S8. Enzymes involved in the tryptophan metabolism of *B. megaterium* NCT-2 from KEGG.** Genes of *B. megaterium* NCT-2 were shown in green boxes.

**Table S1. Plasmids features of *B. megaterium* NCT-2**

| Features | pNCT2-1 | pNCT2-2 | pNCT2-3 | pNCT2-4 | pNCT2-5 | pNCT2-6 | pNCT2-7 | pNCT2-8 | pNCT2-9 | pNCT2-10 |
| --- | --- | --- | --- | --- | --- | --- | --- | --- | --- | --- |
| Size(bp) | 132,087 | 131,764 | 119,712 | 109,637 | 67,346 | 57,761 | 38,703 | 13,449 | 10,257 | 9,625 |
| G+C content (%) | 34.7 | 34.6 | 33.9 | 35.4 | 34.5 | 35.1 | 37.0 | 33.7 | 34.7 | 34.4 |
| Number of protein-coding sequence | 191 | 156 | 159 | 145 | 83 | 63 | 54 | 21 | 18 | 16 |
| Number of RNAs | 0 | 1 | 0 | 0 | 0 | 1 | 20 | 0 | 0 | 0 |
| GenBank accession No. | CP032528.1 | CP032529.1 | CP032530.1 | CP032531.1 | CP032532.1 | CP032533.1 | CP032534.1 | CP032535.1 | CP032536.1 | CP032537.1 |

**Table S2. The functional similarities of *B. megaterium* NCT-2 with 1374 bacterial genomes^*^**

| Organism | Habitat | Temperature | Oxygen | Similarity | Shared  Functions | Functional  Repertoire Size |
| --- | --- | --- | --- | --- | --- | --- |
| *Bacillus megaterium* DSM319 | Soil | Mesophile | Facultative | 90% | 3490 | 3694 |
| *Bacillus megaterium* QM B1551 | Soil | Mesophile | Aerobe | 89% | 3464 | 3720 |
| *Bacillus cereus* biovar anthracis CI | Soil, Host | Mesophile | Aerobe | 54% | 2098 | 3805 |
| *Bacillus cereus* E33L | Host, Soil | Mesophile | Facultative | 54% | 2081 | 3790 |
| *Bacillus* *anthracis* Sterne | Soil | Mesophile | Facultative | 54% | 2086 | 3824 |
| *Bacillus thuringiensis* serovar konkukian 97 27 | Host, Soil | Mesophile | Facultative | 54% | 2090 | 3742 |
| *Bacillus cereus* Q1 | Soil (Oil fields, Soil) | Mesophile | Facultative | 53% | 2075 | 3865 |
| *Bacillus weihenstephanensis* KBAB4 | Soil | Psychrophile (Psychrotolerant) | Facultative | 53% | 2080 | 3845 |
| *Bacillus subtilis* BSn5 | Plants, Soil, Host | Mesophile | Facultative | 53% | 2055 | 3230 |
| *Bacillus anthracis* A0248 | Host | Mesophile | Facultative | 53% | 2061 | 3854 |
| *Bacillus cereus* ATCC 14579 | Soil, Host | Mesophile | Facultative | 53% | 2074 | 3886 |
| *Bacillus subtilis* 168 | Soil | Mesophile | Facultative | 53% | 2055 | 3250 |
| *Bacillus thuringiensis* BMB171 | Host | Mesophile | Aerobe (Microaerophilic) | 53% | 2048 | 3775 |
| *Bacillus subtilis* spizizenii W23 | Soil | Mesophile | Facultative | 52% | 2031 | 3155 |
| *Bacillus thuringiensis* Al Hakam | Host, Soil | Mesophile | Facultative | 52% | 2009 | 3460 |
| *Bacillus atrophaeus* 1942 | Soil | Mesophile | Facultative | 52% | 2014 | 3289 |
| *Bacillus licheniformis* ATCC 14580 | Soil, Host | Mesophile | Facultative | 52% | 2022 | 3189 |
| *Bacillus cereus* B4264 | Host, Soil | Mesophile | Facultative | 52% | 2100 | 4051 |
| *Bacillus cereus* AH187 | Host, Soil | Mesophile | Facultative | 52% | 2094 | 4052 |
| *Bacillus subtilis* spizizenii TU B 10 |  |  |  | 52% | 2026 | 3294 |
| *Bacillus amyloliquefaciens* FZB42 | Rhizosphere-colonizing, Soil, Terrestrial | Mesophile | Aerobe | 52% | 2005 | 2849 |
| *Bacilljjus cereus* G9842 | Soil, Host | Mesophile | Facultative | 52% | 2095 | 4042 |
| *Bacillus anthracis* Ames | Soil | Mesophile | Aerobe | 52% | 2078 | 4031 |
| *Bacillus cereus* AH820 | Soil, Host | Mesophile | Facultative | 52% | 2104 | 4065 |
| *Bacillus cereus* 03BB102 | Soil, Host | Mesophile | Facultative | 52% | 2117 | 4046 |
| *Bacillus anthracis* CDC 684 | Soil, Host | Mesophile | Aerobe | 52% | 2111 | 4060 |
| *Bacillus licheniformis* ATCC 14580 | Soil, Host | Mesophile | Facultative | 52% | 2013 | 3229 |
| *Bacillus anthracis* Ames Ancestor | Soil, Host | Mesophile | Facultative | 52% | 2070 | 3978 |
| *Bacillus amyloliquefaciens* DSM 7 | Soil | Mesophile | Aerobe | 51% | 1981 | 3009 |
| *Bacillus cereus* ATCC 10987 | Dairy isolate, Soil, Host | Mesophile | Facultative | 49% | 2085 | 4250 |
| *Bacillus pumilus* SAFR 032 | Soil, Terrestrial | Mesophile | Aerobe | 49% | 1912 | 2914 |
| *Bacillus pseudofirmus* OF4 | Soil, Terrestrial | Mesophile | Facultative | 47% | 1846 | 3140 |
| *Geobacillus thermodenitrificans* NG80 2 | Fresh water (Fresh water, Oil fields) | Thermophile | Facultative | 47% | 1828 | 2705 |
| *Geobacillus* Y4 1MC1 |  | Thermophile | Facultative | 47% | 1843 | 2795 |
| *Geobacillus thermoglucosidasius* C56 YS93 |  | Thermophile (Hyperthermophile) | Facultative | 47% | 1831 | 2882 |
| *Bacillus cytotoxicus* NVH 391 98 | Soil, Terrestrial | Psychrophile (Psychrotolerant) | Facultative | 47% | 1817 | 3026 |
| *Geobacillus* C56 T3 |  | Mesophile | Facultative | 46% | 1798 | 2664 |
| *Geobacillus* Y412MC61 |  | Thermophile | Facultative | 46% | 1806 | 2699 |
| *Bacillus halodurans* C 125 | Fresh water, Soil | Mesophile | Facultative | 46% | 1802 | 3127 |
| *Geobacillus* Y412MC52 | Fresh water, Hot spring | Thermophile | Facultative | 46% | 1807 | 2698 |
| *Geobacillus kaustophilus* HTA426 | Marine (Deep sea, Marine, Sediment) | Thermophile | Aerobe | 46% | 1803 | 2756 |
| *Bacillus clausii* KSM K16 | Soil | Mesophile | Aerobe | 45% | 1731 | 3040 |
| *Geobacillus* WCH70 |  | Thermophile | Facultative | 45% | 1738 | 2469 |
| *Bacillus coagulans* 36D1 | Soil | Thermophile (Thermotolerant) | Facultative | 44% | 1695 | 2585 |
| *Lysinibacillus sphaericus* C3 41 | Host, Mosquito, Soil | Mesophile | Aerobe | 44% | 1724 | 3472 |
| *Oceanobacillus iheyensis* HTE831 | Soil | Mesophile | Aerobe | 43% | 1681 | 2737 |
| *Bacillus cellulosilyticus* DSM 2522 | Soil | Mesophile | Facultative | 43% | 1684 | 3414 |
| *Brevibacillus brevis* NBRC 100599 | Soil | Mesophile | Aerobe | 42% | 1798 | 4297 |
| *Anoxybacillus flavithermus* WK1 | Fresh water, Hot spring | Thermophile | Facultative | 42% | 1620 | 2329 |
| *Paenibacillus polymyxa* E681 | Rhizosphere | Mesophile |  | 42% | 1643 | 3440 |
| *Paenibacillus polymyxa* SC2 | Rhizosphere | Mesophile |  | 42% | 1673 | 3993 |
| *Bacillus coagulans* 2 6 | Soil | Thermophile | Facultative | 41% | 1607 | 2405 |
| *Paenibacillus* JDR 2 | Host, Wood | Mesophile | Aerobe | 41% | 1666 | 4104 |
| *Paenibacillus* Y412MC10 |  | Mesophile | Facultative | 40% | 1702 | 4238 |

* This table only shows the organisms with the similarity >40%.

**Table S3. Gene cluster involved in nitrogen metabolism of *B. megaterium* NCT-2**

| Location | Strand | Role | Abbrev. | Subsystem |
| --- | --- | --- | --- | --- |
| Chromosome 2156558-2157784 | + | Ammonium transporter | Amt | Ammonia assimilation |
| Chromosome 2508130-2509413 | + | Ammonium transporter | Amt | Ammonia assimilation |
| Chromosome 4041671-4040091 | - | Ferredoxin-dependent glutamate synthase (EC 1.4.7.1) | GOGATF | Ammonia assimilation |
| Chromosome 3102788-3098235 | - | Glutamate synthase [NADPH] large chain (EC 1.4.1.13) | GOGDP1 | Ammonia assimilation |
| Chromosome 3987801-3988598 | + | Glutamate synthase [NADPH] large chain (EC 1.4.1.13) | GOGDP1 | Ammonia assimilation |
| Chromosome 3098218-3096737 | - | Glutamate synthase [NADPH] small chain (EC 1.4.1.13) | GOGDP2 | Ammonia assimilation |
| Chromosome 1165271-1166605 | + | Glutamine synthetase type I (EC 6.3.1.2) | GSI | Ammonia assimilation |
| Chromosome 3583603-3581558 | - | Nitrogen regulation protein NR(I) | NRI | Ammonia assimilation |
| Chromosome 2157799-2158161 | + | Nitrogen regulatory protein P-II | PII | Ammonia assimilation |
| Chromosome 3029532-3027616 | - | Nitric oxide reductase activation protein NorD | DorD | Denitrifying reductase gene clusters |
| Chromosome 3030434-3029544 | - | Nitric oxide reductase activation protein NorQ | DorQ | Denitrifying reductase gene clusters |
| Chromosome 4427681-4425531 | - | Assimilatory nitrate reductase large subunit (EC:1.7.99.4) | NaRas | Nitrate and nitrite ammonification |
| Chromosome 2624008-2625126 | + | Nitrate/nitrite sensor protein (EC 2.7.3.-) | NaNiS | Nitrate and nitrite ammonification |
| Chromosome 4424300-4425481 | + | Nitrate/nitrite transporter | NaNiT | Nitrate and nitrite ammonification |
| Chromosome 4036437-4034023 | - | Nitrite reductase [NAD(P)H] large subunit (EC 1.7.1.4) | NiRas1 | Nitrate and nitrite ammonification |
| Chromosome 4430046-4427701 | - | Nitrite reductase [NAD(P)H] large subunit (EC 1.7.1.4) | NiRas1 | Nitrate and nitrite ammonification |
| Chromosome 4034004-4033678 | - | Nitrite reductase [NAD(P)H] small subunit (EC 1.7.1.4) | NiRas2 | Nitrate and nitrite ammonification |
| Chromosome 751724-752332 | + | Manganese superoxide dismutase (EC 1.15.1.1) | SoD2 | Nitric oxide synthase |
| Chromosome 4831810-4830725 | - | Nitric oxide synthase oxygenase (EC 1.-.-.-) | NOSOx | Nitric oxide synthase |
| Chromosome 1377360-1378574 | + | putative cytochrome P450 hydroxylase | P450h | Nitric oxide synthase |
| Chromosome 3118975-3117764 | - | putative cytochrome P450 hydroxylase | P451h | Nitric oxide synthase |
| Chromosome 3303646-3304878 | + | putative cytochrome P450 hydroxylase | P452h | Nitric oxide synthase |
| Chromosome 4829923-4830372 | + | Nitrite-sensitive transcriptional repressor NsrR | NsrR | Nitrosative stress |
| Plasmids |  | Nitrite-sensitive transcriptional repressor NsrR | NsrR* | Nitrosative stress |

^*^ the gene exists only in *B. megaterium* NCT-2 compared with *B. megaterium* DSM 319 and *B. megaterium* QM B1551
